# Data-Driven Corrections of Partial Lotka–Volterra Models

**DOI:** 10.3390/e22111313

**Published:** 2020-11-18

**Authors:** Rebecca E. Morrison

**Affiliations:** Department of Computer Science, University of Colorado Boulder, 1111 Engineering Drive, Boulder, CO 80309, USA; rebeccam@colorado.edu

**Keywords:** model error, Lotka–Volterra equations, partial models, data-driven model correction, Bayesian calibration and validation

## Abstract

In many applications of interacting systems, we are only interested in the dynamic behavior of a subset of all possible active species. For example, this is true in combustion models (many transient chemical species are not of interest in a given reaction) and in epidemiological models (only certain subpopulations are consequential). Thus, it is common to use greatly reduced or partial models in which only the interactions among the species of interest are known. In this work, we explore the use of an embedded, sparse, and data-driven discrepancy operator to augment these partial interaction models. Preliminary results show that the model error caused by severe reductions—e.g., elimination of hundreds of terms—can be captured with sparse operators, built with only a small fraction of that number. The operator is embedded within the differential equations of the model, which allows the action of the operator to be interpretable. Moreover, it is constrained by available physical information and calibrated over many scenarios. These qualities of the discrepancy model—interpretability, physical consistency, and robustness to different scenarios—are intended to support reliable predictions under extrapolative conditions.

## 1. Introduction

In the realm of computational modeling today, scientists, mathematicians, and engineers investigate, design, optimize, and make predictions and decisions about an incredible multitude of real-world systems. In general, a computational model implements a mathematical model; the mathematical model represents the actual system in question using abstraction and simplification. In this paper, we investigate what happens when common simplifications go too far—resulting in an overly reduced or partial model—and how to account for the discrepancy between this model and the true system of interest. At the same time, these partial models still contain significant deterministic information, and they should not be thrown out entirely. Instead, we augment the partial models with a data-driven correction: we use what we know, and learn the rest.

Partial models are especially common in the context of the generalized Lotka–Volterra (GLV) equations. These equations describe the interactive behavior of any number *S* of different species. The concentration of each species is represented by a variable $x_{i},i=1,\ldots S$; there is one differential equation for each $x_{i}$ whose right-hand side (RHS) includes a linear growth rate term and nonlinear interaction terms. This framework, also called the quasipolynomial form, is canonical in dynamical systems [1] and is used to describe many types of physical systems, including reaction models for chemical kinetics [2], ecological models [3], and epidemiological models [4]. In these applied fields, modelers commonly build a partial model with only s<S species. For example, there are over 50 chemical species thought to be involved in methane combustion [5]; in practice, often merely five to ten species are included [6]. Surprisingly, Kourdis and Belan found that the removal of 90–99% of chemical species can still lead to reliable output [7,8]. SEIR-type epidemiology models include subpopulations of Susceptible, Exposed, Infected, and Recovered (hence the name) humans and the disease carriers (e.g., mosquitoes), while omitting many others such as asymptomatic or hospitalized humans [9], or even cattle or nonhuman primates [10].

*Validation* is the process by which we check that the mathematical model faithfully represents the system in question. While all models should undergo validation, this is especially important when we know our models are incomplete. (*Verification* is the process by which we check that any computation correctly solves the mathematical problems. For example, proper verification procedures include code documentation, unit and regression testing, and solution comparisons against a posteriori error estimates, to name a few. While critical to the success of computational modeling, verification is not a concern of this paper; we assume all computational implementations are correctly documented, implemented, and executed. For more information about verification, see, e.g., [11,12,13].) In its most basic form, validation checks that the model output is consistent with all relevant observations. Statistical techniques that do not require knowledge of the model include, for example, goodness of fit (computing $R^{2}$ values), analysis of residuals between model output and data, and *k*-fold cross-validation [14]. However, a validation process may require a more nuanced procedure, depending on what one plans to do with the model. In [15], Oliver et al. described a sophisticated approach to model validation for predictions of unobservable quantities. Their framework relies on knowledge of the model and system under study, and it takes the behavior of the model over different scenarios into account. In [16], Bayarri et al. described a comprehensive framework for the validation of computer experiments. Their framework includes detailed processes such as determining appropriate domains of model inputs, guarding against overfitting, and accounting for bias in the simulation output. In [17], Farrell-Maupin and Oden described an adaptive method for model calibration and validation using increasingly complex models. In that method, additional richness is only introduced to the model after the simpler version is shown to be invalid. Finally, Jiang et al. implemented a sequential approach to model calibration, validation, and experimental design with the goal of reducing model bias by misspecified and reduced models [18].

Note that all validation procedures rely on access to observations, which should (hopefully) include a description of the associated measurement error. If there is some mismatch between the model and the observations, the source of the discrepancy could either be the model or the observations, or both. Reliable experimental practices and proper data reduction techniques ensure that all observations are reported correctly with quantified measurement uncertainty. In this paper, we assume that any discrepancy between the model and observations is not caused by faulty experimental procedures or reporting. In this way, we may focus on what to do when the model itself causes the discrepancy.

Once validation reveals a discrepancy that cannot be reasonably explained by measurement error, we must improve the model. Consider that the discrepancy is revealed by comparing some set of model output to the corresponding set of observations. If a bias is perceived between the two, a natural first step would be to attach a discrepancy function or stochastic process to the model output, which can then be calibrated to correct the model. In fact, this type of discrepancy model, which we call a response discrepancy model, has been duly investigated, starting with the fundamental work of Kennedy and O’Hagan in 2001 [19]. Since then, the response discrepancy model has been adapted into fields as diverse as climate modeling [20], hydrology [21], and cardiology [22], among many others. Lewis et al. developed an information-theoretic approach to calibrate a low-fidelity model, including a response discrepancy function, from high-fidelity model outputs [23]. This approach is based on Bayesian experimental design to both minimize uncertainty in the low-fidelity model parameters and reduce the number of needed high-fidelity model runs. A response discrepancy model can be useful and relatively quick to develop when one only needs to interpolate between data points.

Recall, however, our goals for computational modeling: to investigate, design, optimize, and make predictions and decisions. To achieve these goals, we must be able to trust the model output beyond a specific calibration scenario; otherwise, we could just rely on observations without need for a model. To this end, we aim to represent the model discrepancy with a discrepancy operator embedded within the model itself, i.e., an embedded discrepancy operator. There are several advantages of an embedded discrepancy operator. First, the operator can be constrained by physical information such as conservation laws, symmetries, fractional concentrations, nonnegativity constraints, and so on. Second, as a function of state variables or other existing model variables, the action of the discrepancy operator is physically interpretable. Third, the operator can be calibrated over many different scenarios, such as initial conditions, boundary conditions, or simulation geometries. Because of these qualities—physical consistency, interpretability, and robustness in different scenarios—an embedded discrepancy model could be valid for extrapolative predictions.

Embedded, or intrusive, approaches have been previously investigated. In [24], Sargsyan et al. allowed for model error by endowing model parameters with random variable expansions. As an approach to model discrepancy, this does not break physical constraints, and the random parameters can be calibrated over many scenarios. However, not all model error can by captured in this way. With complex computational models, missing physics or misspecified physics sometimes causes the discrepancy. This is the situation considered in the current paper. Thus, the discrepancy model becomes part of the model itself, yielding an augmented or enriched model, in which case the specific form of the discrepancy model depends on the modeling context. In [25], the authors investigated this type of inadequacy operator in the context of chemical kinetics for combustion. In this work, we propose and analyze a class of embedded discrepancy operators in the context of the generalized Lotka–Volterra equations, and we show that the error of highly reduced models can be captured by sparse linear operators. For example, a detailed model with 20 species includes 420 parameters where 400 correspond to nonlinear terms, a partial model of four species includes 20 of these, and our discrepancy operator introduces only eight new linear terms.

Although the context is different, the work here is perhaps most similar in philosophy and techniques to that which examines the closure problem of reduced-order fluid dynamics models such as RANS (Reynolds-averaged Navier–Stokes) and LES (large-eddy simulation). For example, Portone et al. developed an embedded operator in [26] for porous media flow models of contaminant transport. Pan and Duraisamy constructed general data-driven closure models for both linear and chaotic systems, including canonical ordinary differential equations (ODEs) and the one-dimensional Burgers equation [27]. Other works describe data-driven closure models using proper orthogonal decomposition [28], Mori–Zwanzig techniques [29], and linear approximations to closure models for the Kuramoto–Sivashinsky equation [30]. In a sense, the current paper presents a type of data-driven “closure model” for partial Lotka–Volterra equations.

The paper is organized as follows. A brief review of the generalized Lotka–Volterra (GLV) equations along with a description of the detailed and partial models is given in Section 2. In Section 3, a class of embedded discrepancy operators and a method for enforcing physics-based constraints are proposed. The details of calibration and validation for the enriched models are given in Section 4. Numerical results are listed in Section 5, and the paper concludes with a discussion in Section 6. Existing techniques to manipulate ordinary differential equations that motivate the proposed discrepancy operators are reviewed in Appendix A.

## 2. Generalized Lotka–Volterra Equations

The generalized Lotka–Volterra equations are coupled ordinary differential equations, used to model the time dynamics of any number of interacting quantities. In particular, the Lotka–Volterra framework allows for linear (growth rate) and quadratic (interaction) terms.

### 2.1. Detailed Models

The objects in the detailed models will be denoted with a $\hat{\;}$ symbol. Let the vector $\hat{x}\in R^{S}$ represent species concentrations. Here, the units of a particular ${\hat{x}}_{i}$ refer to the number of specimens per unit area, but specific units are omitted in this paper. The GLV equations of the detailed model D are written succinctly as:

(1) $$\frac{d\hat{x}}{dt}=D\left(\hat{x}\right)=\mathrm{diag}\left(\hat{x}\right)\left(\hat{r}+\hat{A}\hat{x}\right),$$

where the vector $\hat{r}\in R^{S}$ represents the intrinsic growth rates, and the matrix $\hat{A}\in R^{S\times S}$ collects the interaction rates—that is, the ijth entry of $\hat{A}$, $a_{ij}$, indicates how species *j* affects the concentration of species *i*. The equilibrium solution is ${\hat{x}}_{eq}=-{\hat{A}}^{-1}\hat{r}$.

Since this model is completely determined by the vector $\hat{r}$ and the matrix $\hat{A}$ (modulo initial conditions), we also say that $D=\{\hat{A},\hat{r}\}$. The species included in the detailed model are called the detailed set. The term intraspecific refers to interactive behavior within a particular species (the ${\hat{a}}_{ii}$ are intraspecific terms), while the term interspecific refers to the behavior between two different species (the $a_{ij},i\;\neq j$, are interspecific terms).

### 2.2. Partial Models

The partial model is comprised of all terms involving the *s* species of interest, i.e., by subsampling the detailed one. For example, suppose S=3 and s=2. Then, the detailed model, written out, is

(2a) $$\begin{array}{cc} \frac{d{\hat{x}}_{1}}{dt} & =r_{1}{\hat{x}}_{1}+\left(a_{11}{\hat{x}}_{1}+a_{12}{\hat{x}}_{2}+a_{13}{\hat{x}}_{3}\right){\hat{x}}_{1} \end{array}$$

(2b) $$\begin{array}{cc} \frac{d{\hat{x}}_{2}}{dt} & =r_{2}{\hat{x}}_{2}+\left(a_{21}{\hat{x}}_{1}+a_{22}{\hat{x}}_{2}+a_{23}{\hat{x}}_{3}\right){\hat{x}}_{2} \end{array}$$

(2c) $$\begin{array}{cc} \frac{d{\hat{x}}_{3}}{dt} & =r_{3}{\hat{x}}_{3}+\left(a_{31}{\hat{x}}_{1}+a_{32}{\hat{x}}_{2}+a_{33}{\hat{x}}_{3}\right){\hat{x}}_{3} \end{array}$$

and the partial model is (now without the $\hat{\;}$)

(3a) $$\begin{array}{cc} \frac{dx_{1}}{dt} & =r_{1}x_{1}+\left(a_{11}x_{1}+a_{12}x_{2}\right)x_{1} \end{array}$$

(3b) $$\begin{array}{cc} \frac{dx_{2}}{dt} & =r_{2}x_{2}+\left(a_{21}x_{1}+a_{22}x_{2}\right)x_{2}. \end{array}$$

Likewise, the partial model is referred to as P, so

(4) $$\frac{dx}{dt}=P\left(x\right)=\mathrm{diag}\left(x\right)\left(r+Ax\right),$$

and P={A,r}. Here, the equilibrium solution is $x_{eq}=-A^{-1}r$.

In this example, the growth rate vectors and interaction matrices are

(5) $$\hat{A}=\left[\begin{array}{ccc} a_{11} & a_{12} & a_{13}\\ a_{21} & a_{22} & a_{23}\\ a_{31} & a_{32} & a_{33} \end{array}\right],\;\hat{r}=\left[\begin{array}{c} r_{1}\\ r_{2}\\ r_{3} \end{array}\right],\;A=\left[\begin{array}{cc} a_{11} & a_{12}\\ a_{21} & a_{22} \end{array}\right],\;r=\left[\begin{array}{c} r_{1}\\ r_{2} \end{array}\right].$$

The species included in the partial model are called the partial set and sometimes also the remaining species—that is, remaining after a reduction process.

### 2.3. Defining the Scope

As the objective of this paper is to understand model discrepancy in the context of partial GLV models, we must define the scope of this context. There are a few considerations to keep in mind. First, note that, as explained above, the partial model considered here follows immediately from the detailed model. Thus, when determining the scope of models under investigation, it suffices to determine the detailed model(s). Then, given a detailed model, we investigate all possible partial models from s=1 to s=S−1.

Second, the GLV equations encompass an infinite number of specific models, or model realizations, as *S* can be any integer ≥2, and the entries of $\hat{A}$ and $\hat{r}$ can be, in theory, any real numbers. Moreover, any two GLV models, determined by a specific $\hat{A}$ and $\hat{r}$, may behave differently from one another. At the most specific extreme, all model parameters are fixed, yielding a single fixed pair of detailed and partial models, and we could then investigate the model discrepancy therein. At the most general extreme, many models are supplied via highly unconstrained realizations of the model parameters, and we could hope to thus discover highly general results about the model discrepancy. In this paper, by aiming somewhere in between these two extremes, we examine a moderately general random class of LV models. This class is determined by specifying appropriate distributions for the entries of $\hat{A}$ and $\hat{r}$.

Third, since this is an initial exploration into representing model discrepancy in the GLV context, let us narrow the scope in order to examine well-behaved models, i.e., those with stable equilibria. We focus on symmetric interaction matrices $\hat{A}$ with negative entries. This constraint says that all interactions between and within species are competitive, not cooperative. Then, the matrix $\hat{A}$ can be stabilized by making its diagonal entries larger in magnitude than the sum of off-diagonal entries in the same row (or column), ensuring diagonal dominance and thus negative eigenvalues. Here, we consider models whose interaction matrices are symmetric, diagonally dominant, and have negative entries. These restrictions (or similar, e.g., that $\hat{A}$ be negative definite) are common assumptions in mathematical ecology studies; see, e.g., [3,31,32]. The distributional form characterizing entries of $\hat{A}$ and $\hat{r}$ are given in the following subsection. Then, in Section 5, specific models are sampled and analyzed through numerical examples.

### 2.4. Creating the GLV Detailed and Partial Models

In this subsection, the information above is summarized and refined algorithmically. Algorithm 1 generates a realization of a detailed model, and Algorithm 2 provides the corresponding partial model. Recall that we use $\hat{\;}$ to differentiate between the two and denote a quantity of the detailed model.
**Algorithm 1** Generating a realization of the detailed model.1:Initialize *S*2:Sample $B_{ij}∼\log N\left(0,\sigma_{B}^{2}\right),1\leq i<j\leq S$3:Set $B_{ji}=B_{ij}$4:Sample $C_{ii}∼\log N\left(0,\sigma_{C}^{2}\right)+\sum_{k\neq i}B_{ki},1\leq i\leq S$5:Set interaction matrix $\hat{A}=-\left(B+C\right)$6:Set growth rate vector $\hat{r}=\max\left\{C\right\}1_{S}$7:**return**$D=\{\hat{A},\hat{r}\}$**Algorithm 2** Subsampling the partial model.1:Initialize s<S, *D*2:Set *A* as submatrix: $A={\hat{A}}_{1:s,1:s}$3:Set *r* as subvector: $r={\hat{r}}_{1:s}$4:**return**P={A,r}

Without loss of generality, we simply choose the first *s* species in Algorithm 2, as the detailed set follows no special or implicit ordering. Note that existence of a stable equilibrium of the partial model follows directly from that of the detailed model.

## 3. Enriched GLV Model

Previous work shows how a set of *S* coupled Lotka–Volterra equations can be converted to a set of *s* equations, s<S, using algebraic substitutions and/or integration [33]. The resulting equations will either need to depend on higher derivatives of the remaining species or on their complete time history—that is, the exact dynamics from the detailed model may be written only in terms of the partial set:

(6) $$\frac{dx}{dt}=F\left(x,\dot{x},\ddot{x},\ldots ,K\left(x\right)\right),$$

where $\dot{x}$ is the first derivative of x, $\ddot{x}$ the second derivative, and so on, and K(x) represents some memory kernel. Two such manipulations are reviewed in Appendix A. This motivates an approximation of F with the available partial model P and a discrepancy model Δ that is a function of either the derivatives or memories of the remaining variables—that is, we seek a model for the partial set of variables as:

(7) $$\frac{dx}{dt}\approx P\left(x\right)+\Delta \left(x,\dot{x},\ddot{x},K\left(x\right)\right).$$

The above may be reminiscent of Takens’s theorem [34], in which a dynamical system is reconstructed from (delayed) observations of the system. However, the two approaches differ fundamentally: here, the LHS derivatives are restricted to those of the partial set, i.e., a subset of the original variables, but that is not true in a delay embedding.

We now propose a particular form of Δ.

### 3.1. Linear Embedded Discrepancy Operator

Recall that the detailed model is

(8) $$\frac{d\hat{x}}{dt}=D\left(\hat{x}\right)=\mathrm{diag}\left(\hat{x}\right)\left(\hat{r}+\hat{A}\hat{x}\right).$$

and the partial model is

(9) $$\frac{dx}{dt}=P\left(x\right)=\mathrm{diag}\left(x\right)\left(r+Ax\right).$$

We initially propose an enriched model E, linear in $\left(x,\dot{x}\right)$, of the form

(10a) $$\begin{array}{cc} \frac{dx}{dt} & =E(x,\dot{x}) \end{array}$$

(10b) $$\begin{array}{cc} \; & =P\left(x\right)+\mathrm{diag}\left(x\right)\delta_{0}+\mathrm{diag}\left(\dot{x}\right)\delta_{1} \end{array}$$

(10c) $$\begin{array}{cc} \; & =P\left(x\right)+\Delta \left(x,\dot{x}\right), \end{array}$$

where $\delta_{0}={\left(\delta_{10},\delta_{20},\ldots ,\delta_{s0}\right)}^{T}$ and $\delta_{1}={\left(\delta_{11},\delta_{21},\ldots ,\delta_{s1}\right)}^{T}$. The subscripts on each $\delta_{ij}$ are chosen so that *i* indicates that this coefficient appears in the RHS of the variable $x_{i}$, and *j* indicates that this coefficient is multiplying the *j*th derivative of $x_{i}$.

A major advantage of an embedded operator, as opposed to a response discrepancy model, is that the operator can be constrained by any available information about the physical system. In this simple example, we do have some information about the system that implies constraints on the introduced discrepancy parameters $\delta_{0},\delta_{1}$. First, we make the modeling ansatz that these discrepancy parameters should not depend explicitly on time. A result of this ansatz is then that the parameters be constrained independently. We also (assume that we) know that all interspecific interactions are competitive. In particular, note that $a_{ij}x_{i}x_{j}<0$ because $a_{ij}<0$ and $x_{i},x_{j}\geq 0$. Thus, we enforce that $\Delta_{i}\left(x_{i},{\dot{x}}_{i}\right)\leq 0$. Thus, specific information about the high-fidelity physical system implies the following constraints:

- We know $x_{i}\geq 0$ which implies $\delta_{i0}\leq 0$.
- The constraint on $\delta_{i1}$ is slightly less clear since the sign of ${\dot{x}}_{i}$ could be positive or negative. Thus, we could set $\delta_{i1}={\tilde{\delta }}_{i1}\mathrm{sgn}\left({\dot{x}}_{i}\right)$, where ${\tilde{\delta }}_{i1}\leq 0$. Equivalently, we can write the discrepancy as
  (11) $$\Delta_{i}\left(x_{i},{\dot{x}}_{i}\right)=\delta_{i0}x_{i}+\delta_{i1}\left|{\dot{x}}_{i}\right|.$$
  Then, set $\delta_{i1}\leq 0$ and the constraint is satisfied.

Because of this final constraint, the discrepancy operator is no longer linear in $\dot{x}$, but rather in $|\dot{x}|$. We still refer to such a formulation as linear (precedence for this use of linear is found in [35]). Thus, we amend the above enriched model in lines (10a)–(10c) as

(12a) $$\begin{array}{cc} \frac{dx}{dt} & =E(x,|\dot{x}|) \end{array}$$

(12b) $$\begin{array}{cc} \; & =P\left(x\right)+\mathrm{diag}\left(x\right)\delta_{0}+\mathrm{diag}(|\dot{x}\left|\right)\delta_{1} \end{array}$$

(12c) $$\begin{array}{cc} \; & =P\left(x\right)+\Delta (x,|\dot{x}|). \end{array}$$

Finally, the introduced discrepancy parameters $\delta_{0},\delta_{1}$ are calibrated, using observations of species concentrations generated by the detailed model. Indeed, the strength of the embedded operator approach stems from two properties: (1) the ability to constrain the formulation by available physical information, and (2) the ability to leverage information from the detailed system by calibrating the model discrepancy parameters. Moreover, we calibrate over a range of initial conditions, denoted as $\phi_{i},i\;=1,\ldots ,n_{\phi_{c}}$. Note that we also validate over a range of $n_{\phi_{v}}$ initial conditions $\phi_{i},i\;=n_{\phi_{c}}+1,\ldots ,n_{\phi }$ so that $n_{\phi_{c}}+n_{\phi_{v}}=n_{\phi }$. Each $\phi_{i}$ specifies the species initial concentrations:

(13) $$\phi_{i}=\left(x_{1}\left(0\right),x_{2}\left(0\right),\ldots ,x_{s}\left(0\right)\right),\;i=1,\ldots ,n_{\phi }.$$

By calibrating with observations from all $n_{\phi_{c}}$ scenarios, the goal is to build a more robust discrepancy model that is valid over several scenarios instead of only calibrated to a very specific dataset. This property of the model discrepancy construction further allows for the possibility, at least, that such an enriched model could be used in extrapolative conditions, such as a prediction in time, or in scenarios given by different initial conditions.

Note that the actual observations used to calibrate the parameters are specified in Section 4, along with the particulars of the calibration itself. We have tried to separate what is essential to the formation of the discrepancy operator from the calibration details, which could reasonably change based on the example at hand.

### 3.2. Equilibrium and Stability of the Enriched Models

The equilibrium solution of the enriched model is

(14) $$x_{eq}=-A^{-1}\left(r+\delta_{0}\right).$$

Thus, the parameters $\delta_{0}$, which act linearly on the state x, directly control the equilibrium solution. The stability of the enriched model is less obvious because of the absolute values; here, we conjecture that the models are indeed stable.

To see why, first consider an example enriched system of just one variable *x*:

(15) $$\dot{x}=x-x^{2}-\frac{1}{2}\left|\dot{x}\right|.$$

Depending on the sign of $\dot{x}$, this becomes one of the following logistic equations

(16a) $$\begin{array}{cc} \dot{x} & =2x\left(1-x\right),\;\dot{x}<0 \end{array}$$

(16b) $$\begin{array}{cc} \dot{x} & =\frac{2}{3}x\left(1-x\right),\;\dot{x}>0. \end{array}$$

**Figure 1:**
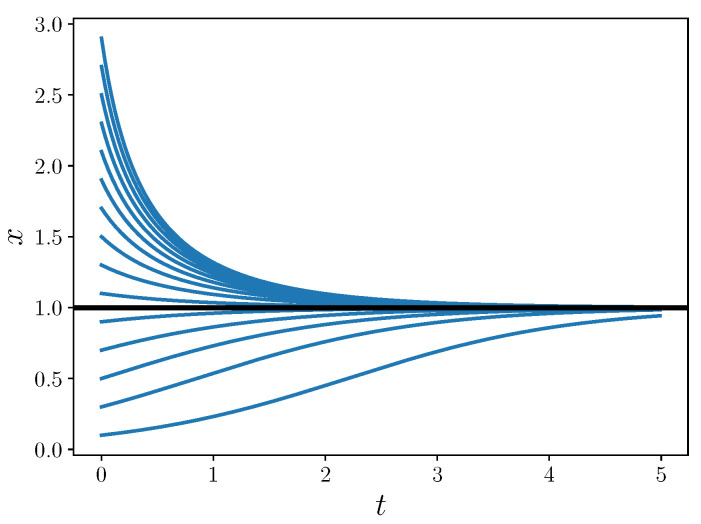
Solutions to the logistic equation. The thick black line shows the x=1 phase line.

The logistic equations admit solutions of the qualitative nature shown in Figure 1.

Importantly, the sign of $\dot{x}$ never changes over any solution curve. Given the initial condition, we could in fact solve the same system without the absolute value by choosing either (16a) or (16b); both reach stable equilibrium. Thus, the presence of the absolute value in this example does not affect the stability of the system.

In general, the signs of the derivatives may change. However, we conjecture that the derivatives of all species do not change sign after a given point in time, say $t^{*}$ (as seen in the numerical examples in Section 5). In this case, the enriched differential equation for $x_{i}\left(t\right),t>t^{*}$ is

(17) $${\dot{x}}_{i}=\lambda_{i}\left(\left(r_{i}+\delta_{i0}\right)x_{i}+\sum_{j=1}^{s}a_{ij}x_{i}x_{j}\right)$$

where

(18) $$\lambda_{i}=\left\{\begin{array}{c} \frac{1}{1+\delta_{i1}},\;{\dot{x}}_{i}\left(t\right)<0\;\;\forall t>t^{*}\\ \frac{1}{1-\delta_{i1}},\;{\dot{x}}_{i}\left(t\right)>0\;\;\forall t>t^{*}. \end{array}\right.$$

The above does reach a stable equilibrium, as $\lambda_{i}$ simply scales the overall dynamics and the interaction matrix *A* (still) determines the stability of the system.

A more rigorous analysis of stability for these systems will be addressed in future work. For now, we note that differential equations with absolute value terms have been treated in the literature. In particular, Khan and Barton showed that, for ODEs whose RHS are a composition of analytic and absolute value functions of the state variables, the arguments of the absolute values change sign finitely many times in any finite duration [36], while Barton et al. provided a theoretical and computational framework for evaluating nonsmooth derivatives called lexicographic directional derivatives [37]. Finally, Oakley demonstrated that certain second-order differential equations with absolute values admit solutions of sets of related linear differential equations [35].

### 3.3. Proof of Concept: Linear Embedded Discrepancy Operator, S=2,s=1

As an initial proof of concept, consider the S=2,s=1 case. The detailed model is

(19) $$\begin{array}{c} D=\left(\hat{A},\hat{r}\right)=\left(\left[\begin{array}{cc} -3 & -1\\ -1 & -2 \end{array}\right],\;\left[\begin{array}{c} 5\\ 3 \end{array}\right]\right) \end{array}$$

and the partial model is simply $P=\left(A,r\right)=\left(a_{11},r_{1}\right)=\left(-3,5\right)$. In this case, the exact discrepancy is $-x_{2}x_{1}$, and we aim to approximate the effect of this term with

(20) $$\Delta_{1}\left(x_{1},{\dot{x}}_{1}\right)=\delta_{10}x_{1}+\delta_{11}\left|{\dot{x}}_{1}\right|.$$

**Figure 2:**
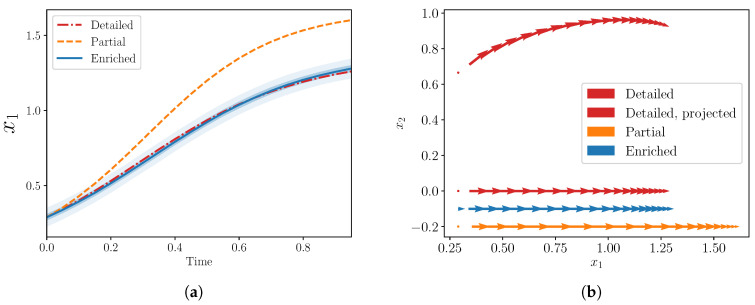
(**a**) Trajectories of $x_{1}$ from detailed, partial, and enriched models for a simple example with S=2, s=1. (**b**) Phase diagrams from the same three models. The 1D derivatives from the enriched model approximately match the projection from the 2D detailed case onto the $x_{1}$-axis. Note that the 1D enriched and partial derivatives are plotted below the projected detailed case for visualization purposes (along lines $x_{2}=-0.1,-0.2$).

Calibration yields posterior mean values of ${\bar{\delta }}_{10}\approx -0.837$ and ${\bar{\delta }}_{11}\approx -0.0224$; further calibration details are deferred to the next section. The three models—detailed, partial, and enriched—are shown in Figure 2a; excellent agreement between the detailed and enriched models is achieved.

We also show the phase diagram of the three models in Figure 2b including the 2D phase diagram from the detailed model projected onto the $x_{1}$-axis. The recovered derivatives of the enriched model approximately match this projection quite well. Analogous plots are difficult to visualize in higher dimensions, but in this low-dimensional case, this projection may provide some intuition about why the enriched model behaves like the detailed model.

### 3.4. Other Possible Formulations

There are a number of related possible formulations of the model discrepancy. Some options are the following:

1. An affine expression up to the *N*th derivative:
  (21) $$\Delta_{i}=\mu_{i}+\sum_{j=0}^{N}\delta_{ij}\frac{d^{j}}{dt^{j}}\left(x_{i}\right).$$
2. A quadratic expression up to the *N*th derivative. Let
  $$q=\left\{\frac{d^{0}}{dt^{0}}\left(x_{1}\right),\ldots ,\frac{d^{N}}{dt}\left(x_{1}\right),\ldots ,\frac{d^{0}}{dt^{0}}\left(x_{s}\right),\ldots ,\frac{d^{N}}{dt^{N}}\left(x_{s}\right)\right\}.$$
  Then
  (22) $$\Delta_{i}=\mu_{i}+\sum_{i,j}^{s(N+1)}\delta_{ij}\left(q_{i}q_{j}\right).$$
3. A memory expression, such as:
  (23) $$\Delta_{i}\left(t\right)=\mu_{i}+\beta_{i}\int_{s=0}^{t}x_{i}\left(s\right)ds$$
  for some $\beta_{i}\in R$.

Each of the above formulations includes an affine term $\mu_{i}$. Whether or not such a constant term would be advantageous when all the missing dynamics terms are state-dependent is not immediately clear.

Of course, one could also propose some combination of the above formulations as an embedded discrepancy operator. Investigating the numerical advantages and limitations of many such discrepancy operators is beyond the scope of the current paper. For now, numerical results are presented in Section 5 about the proposed linear embedded discrepancy operator, as described in Section 3.1.

## 4. Calibration and Validation

This section contains all relevant details about the calibration and validation processes. First, for both of these, it is necessary to know what observations are available.

### 4.1. The Observations

The datasets used to calibrate and validate the discrepancy model include observations from the detailed model trajectories of the *s* species included in the partial model. From each trajectory, *T* observations are taken, and there is a new trajectory for each initial condition ϕ, so that the observations can be summarized as

(24) $$O=\left\{y_{ijk}\right\},i=1,\ldots ,s;j=1,\ldots ,T;k=1,\ldots ,n_{\phi }$$

where $y_{ijk}$ is the observation of $x_{i}\left(t_{j}\right)$ given the initial condition $\phi_{k}$. This observed value $y^{*}$ is given by the true value $y^{t}$ with additive measurement error ϵ:

(25) $$y^{*}=y^{t}+\epsilon ,$$

where the distribution of measurement error is normal: $p_{\epsilon }=N\left(0,\sigma_{\epsilon }^{2}\right)$.

Finally, this set of observations is partitioned into two sets, one for calibration and the other for validation. Let us partition as follows:

(26) $$\begin{array}{cc} \mathrm{Calibration}\;\mathrm{data}:\; & O_{c}=\left\{y_{ijk}\right\},i=1,\ldots ,s;j=1,\ldots ,T;k=1,\ldots ,n_{\phi_{c}} \end{array}$$

(27) $$\begin{array}{cc} \mathrm{Validation}\;\mathrm{data}:\; & O_{v}=\left\{y_{ijk}\right\},i=1,\ldots ,s;j=1,\ldots ,T;k=n_{\phi_{c}}+1,\ldots ,n_{\phi }. \end{array}$$

That is, $n_{\phi_{c}}$ initial conditions are used for calibration, and the remaining $n_{\phi_{v}}$ are designated for validation, where $n_{\phi_{c}}+n_{\phi_{v}}=n_{\phi }$.

### 4.2. Calibration Details

The calibration is done using a Bayesian approach, and the details of the calibration problem are as follows.

- **Prior:** We set uniform prior distributions on the discrepancy parameters θ:
  (28) $$\begin{array}{cc} p(\theta ) & =\prod_{\overset{i=1,\ldots s}{j=0,1}}p\left(\delta_{ij}\right), \end{array}$$
  where
  (29) $$\begin{array}{cc} p(\delta_{ij}) & =U(-100,0)\;i=1,\ldots ,s;j=0,1. \end{array}$$
  (One might expect a negative lognormal distribution for these priors, and this was in fact the first choice. However, the uniform priors performed much better during the sampling process, and all of the parameter chains in Markov Chain Monte Carlo simulations were well-contained by the uniform bounds. Why the lognormal priors led to poor mixing will be investigated further in future work.)
- **Likelihood:** The likelihood is determined by the measurement error:
  (30) $$p\left(O_{c}|\theta \right)=\prod_{l=1,\ldots ,|O_{c}|}p_{\epsilon }\left(y_{l}-y_{l,E}\right)$$
  where the observations have been re-indexed from 1 to $|O_{c}|$ (to avoid triple subscripts here) and $y_{l,E}$ is the corresponding model output from the enriched model E.
- **Posterior:** Given the prior and likelihood distributions above, the posterior distribution follows as:
  (31) $$p\left(\theta |O_{c}\right)\propto p\left(O_{c}|\theta \right)p\left(\theta \right).$$

Specifically, the calibration is performed according to the Delayed Rejection Adaptive Metropolis (DRAM) method, introduced in [38] and implemented in the statistical library QUESO [39].

### 4.3. Validation Metric

Next, we must define an appropriate quantitative validation metric. First, we quantify the consistency between the enriched model output and the corresponding observation. We compute how probable the observation is as a realization of the model output. The probability of observing some $y^{*}$, given the data $O_{c}$, is

(32) $$p\left(y^{*}|O_{c}\right)=\int_{y^{t}}p_{\epsilon }\left(y^{t}-y^{*}\right)\left(\int_{\theta }p\left(y^{t}|\theta \right)p\left(\theta |O_{c}\right)d\theta \right)dy^{t}.$$

We can compare this probability to the rest of possible model outputs. In particular, we are interested in how much of the distribution corresponds to model outputs less likely than the one above in (32). This amount is exactly given by the γ-value, as defined in [15]:

(33) $$\gamma_{y^{*}}=\int_{y\in S}p\left(y|O_{c}\right)dy$$

where $S=\{y:p\left(y|O_{c}\right)\leq p\left(y^{*}|O_{c}\right)\}$. Note that a low γ-value implies that the observation is less probably an outcome of this model than most possible outcomes. In contrast, values that are not low demonstrate consistency between the model and observation. In this work, we compute the fraction of γ-values below a given threshold τ. For a more thorough introduction to γ-values and discussion of their use in model validation, see [15], and for another example of this used in practice as a validation metric, see [25].

**Figure 3:**
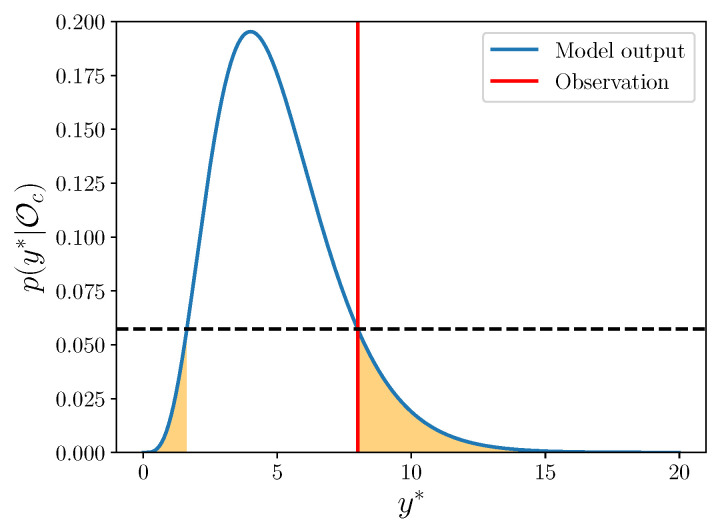
The γ-value corresponds to the shaded area. The dashed horizontal line simply shows the *y*-axis value where the observation crosses the model output density, so that we integrate over the set for which the density is lower than this value.

An example of the area corresponding to this integral is given in Figure 3. In this work, we compute the integrals with a Monte Carlo approach [40].

## 5. Numerical Results

We now present the numerical performance of the proposed linear embedded discrepancy operator described in Section 3.1. All code—to run forward and inverse problems, generate data, and postprocess—is available here: github.com/rebeccaem/enriched-glv [41].

### 5.1. Results for One Realization of the Detailed Model

First, let us examine results for a single detailed and partial model. The detailed model is generated according to Algorithm 1, with the following values:

(34) $$S=10,\;\sigma_{B}^{2}=1,\;\sigma_{C}^{2}=1.$$

Then, the partial model is generated according to Algorithm 2 with s=4. In this example, the observations from the detailed model are taken so that $n_{\phi_{c}}=3$, $n_{\phi_{v}}=3$, T=10, and $\sigma_{\epsilon }^{2}=0.001$. The entries of each initial condition vector $\phi_{i}$ are generated randomly from a lognormal distribution, logN(0,1). Note that 90 parameters are omitted during reduction, while only eight are introduced during enrichment.

**Figure 4:**
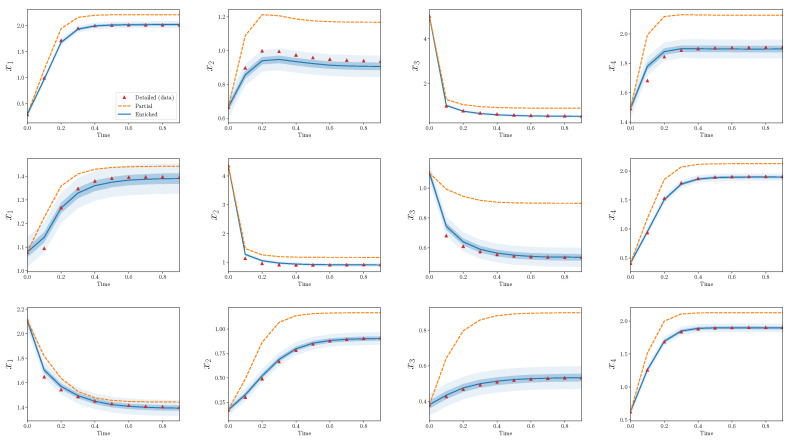
Partial and enriched models, compared to observations, over three calibration scenarios, S=10,s=4. Note that 90 parameters are omitted during reduction, while only eight are introduced during enrichment.

Figure 4 shows trajectories for calibration scenarios from the three models: detailed, partial, and enriched. The 50% and 95% quantiles are plotted for the enriched model output. There is an obvious discrepancy between the output from the detailed and partial models, and the enriched model is able to capture the bulk of this discrepancy. Nearly all of the observations from the detailed model are contained within the model output bounds from the enriched model.

**Figure 5:**
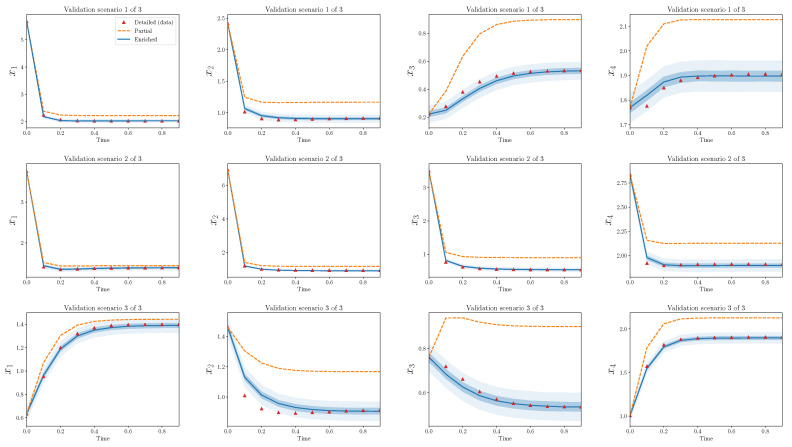
Partial and enriched models, compared to observations, over three validation scenarios. S=10,s=4.

Figure 5 shows the same results, but for validation scenarios. Recall that these observations have not been used to calibrate the discrepancy operator. The output of the enriched model, at least to the eye, appears decent. The enriched model is greatly improved in comparison to the partial model alone and, similarly to the calibration scenarios, captures the bulk behavior of the detailed model in the validation scenarios.

**Figure 6:**
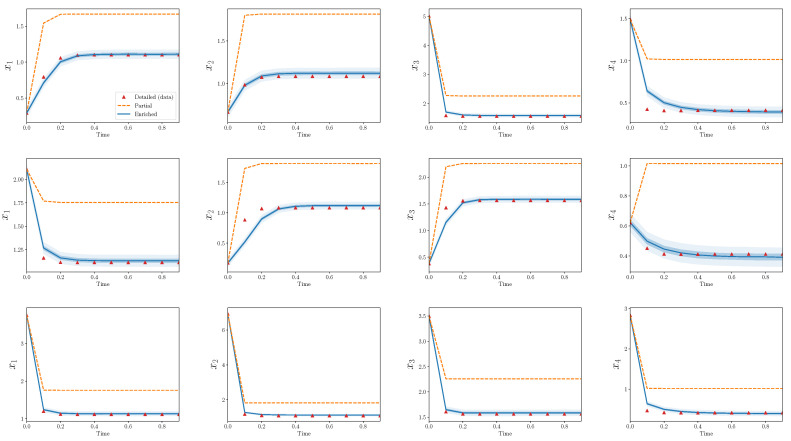
Partial and enriched models, compared to observations, over three calibration scenarios. S=20,s=4. Note that 400 parameters are omitted during reduction, while only eight are introduced during enrichment.

**Figure 7:**
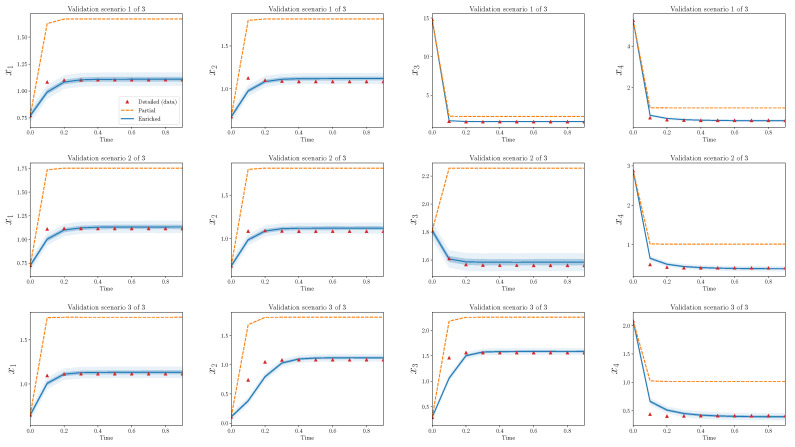
Partial and enriched models, compared to observations, over three validation scenarios. S=20,s=4.

Figure 6 and Figure 7 show analogous plots for S=20, s=4. In this case, 400 parameters are omitted during reduction, while only eight are introduced during enrichment.

In the above cases, there are a few observations which lie outside the predicted bounds of the enriched model. This problem must be addressed more carefully with a quantitative validation process as described in Section 4. Additionally, these results only show the performance of the discrepancy operator for a particular *S* and *s* and a single realization of (D,P). The agreement between trajectories from detailed and partial models for different choices of (D,P) are qualitatively similar, but some interesting differences appear by varying *s* with respect to *S*. In the next subsections, these statements are made more precise.

### 5.2. Results for Many Realizations of the Detailed Model

We examine the performance of the proposed discrepancy model in the context of random forward models. To this end, three relevant concepts are detailed below.

**Figure 8:**
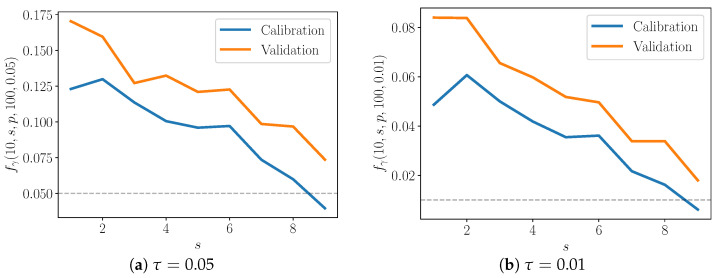
Average fraction of γ-values below given threshold. S=10.

**Figure 9:**
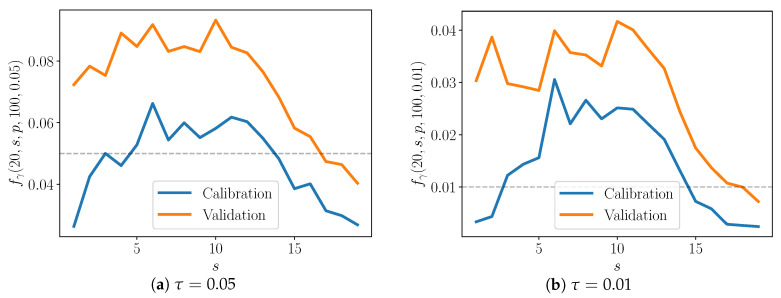
Average fraction of γ-values below given threshold. S=20.

1. We quantify the average performance of the discrepancy models. In this sense, we compute these γ-values for trajectories from $n_{M}$ realizations of detailed models, where $n_{M}\gg 1$.
2. Note γ-values are computed with two types of data: calibration and validation data. To refer to these two types of data, we will use the variable p={c,v}, so that p=c denotes calibration data and p=v denotes validation data. We must check how well the enriched model performs both in terms of the data that has been used to calibrate it, and also in terms of data that has not. Both types are shown in Figure 8 and Figure 9.
3. Finally, let us examine how well the discrepancy operators perform for different pairs (S,s). We fix $S,s,p,n_{M}$ and then compute γ-values for all type *p* observations over $n_{M}$ models, for a particular pair (S,s). Call this set of γ-values $\Gamma (S,s,p,n_{M})$. Now let $Q\left(S,s,p,n_{M},\tau \right)=\left\{\gamma_{i}:\gamma_{i}<\tau ,\gamma_{i}\in \Gamma \left(S,s,p,n_{M}\right)\right\}$. Then, the fraction of γ-values below the threshold τ is:
  (35) $$f_{\gamma }\left(S,s,p,n_{M},\tau \right)=\frac{\left|Q\right|}{\left|\Gamma \right|}.$$
  For example, if we want to compute $f_{\gamma }$ for all calibration data over $n_{M}$ model realizations, the denominator above is $\left|\Gamma \right|=sTn_{\phi_{c}}n_{M}$. The value $f_{\gamma }$ is plotted in Figure 8 and Figure 9, and *S* is fixed at 10 and 20, respectively. Along the *x*-axis, *s* ranges from 1 to S−1. The results for two values of τ—0.05 (shown in Figure 8a and Figure 9a) and 0.01 (shown in Figure 8b and Figure 9b)—are also shown.

Let α=s/S. In the case that the model truly does represent the data-generating process and in the limit of infinite observations, then this fraction of γ-values below the threshold is equal to the threshold itself—that is,

(36) $$\lim_{n_{M}\to \infty }f_{\gamma }\left(S,s,p,n_{M},\tau \right)=\tau ,$$

when the model is a true match to the data-generating process. Indeed, $f_{\gamma }\left(10,s,c,100,\tau \right)$ approaches τ as α approaches 1 (Figure 8). This suggests that the enriched model is better able to capture the behavior of the detailed one as more species are included in the partial model, as one might expect.

Interestingly, in the S=20 case, $f_{\tau }$ peaks somewhere in the middle of the plot, when α≈0.5 (Figure 9). In other words, the enriched model is poorest for moderate α, and performs best as α approaches 1. Consider that when α is low, only a few species are included in the partial model relative to the detailed one, but also consider that the discrepancy model has only those few species to modify. When α is close to one, the partial model already includes much of the detailed model, and the discrepancy model must only fill a small gap between the two. For moderate α, however, there are neither of these advantages—the discrepancy model must account for the behavior of a large enough number of species, but the partial model is still significantly lacking compared to the detailed model.

At the same time, the S=10 plots do not exhibit the above behavior. Note that the S=20 cases appear to reach equilibrium more quickly that those with S=10; the time to equilibrium may influence the shape of curves in Figure 8 and Figure 9. Future work will include extensive numerical testing to better understand these results.

### 5.3. Relative Model Complexity

**Figure 10:**
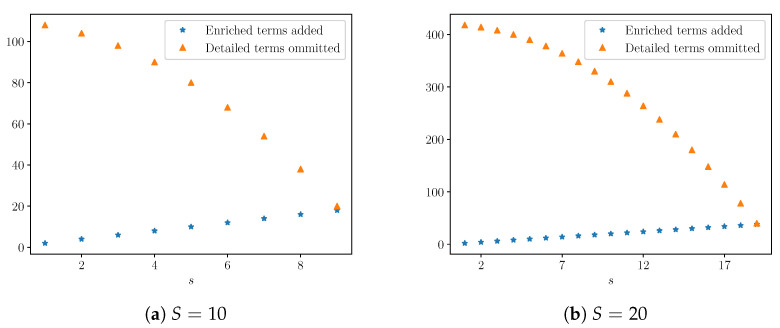
Comparison of number of terms added by the enriched model and terms omitted from the detailed model.

A good discrepancy model should not overfit the data, and the best discrepancy model would be rich enough to capture the relevant behavior of the detailed model without adding unnecessary complexity. Although there are different ways one might measure complexity, here, we measure the number of terms introduced in the enriched model (2s) compared to those omitted from the detailed model. These omitted terms include the $S^{2}-s^{2}$ interspecific and intraspecific interaction terms, as well as the (S−s) growth rate terms. (Note that the number of terms introduced is equal to the number of enriched model parameters.) For the cases S=10,20, the absolute values are shown in Figure 10.

**Figure 11:**
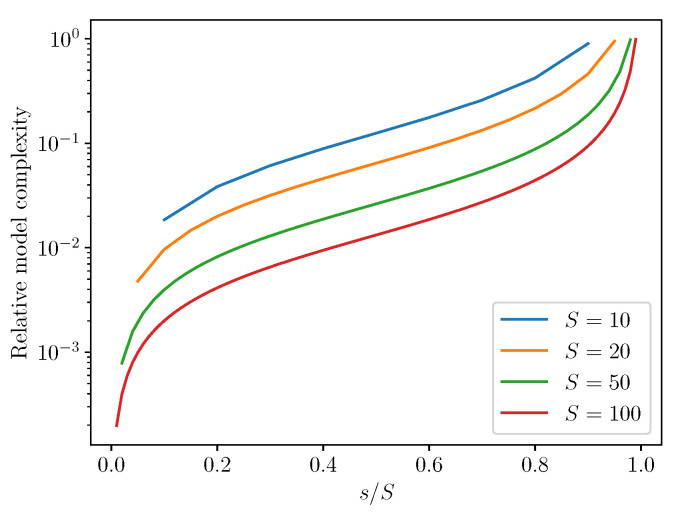
Enriched model complexity, measured as the ratio of enriched model terms added to detailed model terms omitted.

In Figure 11, this information is presented as a ratio of terms added relative to terms omitted for various values of *S*. We call this ratio the relative model complexity.

The relative model complexity is plotted as s/S varies from 1/S to (S−1)/S for a few different values of *S*. These include the two cases presented here (S=10,20). We also show the relative model complexity for two higher values of *S*, namely S=50 and S=100. One might be interested in how this type of model complexity would scale for much larger systems. Moreover, if one knew a priori the true value of *S* for some system, one could balance the effectiveness of the enriched model (as measured by $f_{\tau }$) against its relative model complexity.

Strikingly, the enriched models introduce many fewer terms than what the partial models omit. For example, in the two specific forward models shown in Figure 4, Figure 5, Figure 6 and Figure 7, the relative model complexity is less than 0.1, yet the enriched model and observations do show surprisingly high consistency.

## 6. Conclusions

This study is an initial step toward representing model discrepancy in nonlinear dynamical systems of interacting species. The proposed discrepancy model here is a linear operator embedded within the differential equations. The particular form is motivated by circumstances in which a set of differential equations can be converted to a set of fewer equations; in this decoupling process, more information must be introduced about the remaining set, such as memory or higher derivatives. In this work, the discrepancy model is similarly constructed by introducing more information about the partial set, namely as a linear operator which acts on the remaining variables and (the absolute values of) their first derivatives.

We can examine the performance of the enriched models over two regimes: equilibrium and transient dynamics. The introduced parameters $\delta_{0}$ act on the state variables, directly affect the equilibrium solution, and seem to be sufficient, as the enriched models typically recover equilibria of the detailed models. On the other hand, the parameters $\delta_{1}$ act on the derivatives of the state, provide a type of overall scaling of the dynamics, and give an improvement but not a total correction; the enriched models recover much of the transient dynamics, but certainly not all of the discrepancy for every combination of (S,s). While the performance in the transient regime could be improved, the linear embedded discrepancy operators show promise as discrepancy models, even in scenarios that extrapolate over initial conditions. The results also bring up many new questions.

For example, what is the effective dimension of the missing dynamics of the partial model? In other words, how many (and which) new random variables need to be introduced to effectively (i.e., within some tolerance) capture the error of the partial model? The initial results here suggest that the discrepancy between the partial and detailed models can, under some conditions, be adequately described with a relatively small number of discrepancy variables and parameters. An outstanding question is whether or not some estimate of this effective discrepancy dimension can be found a priori. Certainly, such an estimate would heavily rely on given knowledge of the detailed and partial models.

Another avenue to explore is the design and analysis of more elaborate discrepancy representations in the generalized Lotka–Volterra setting, including those with second (or higher) derivatives, memory, nonlinear terms, or some combination of these. Of course, a trade-off exists between the richness of the discrepancy representation and the computational expense of both the forward and inverse problems.

Finally, the detailed models (and thus also partial models) investigated here are quite simple; the interaction matrices are negative definite, diagonally dominant, and symmetric, with off-diagonal entries sampled from identical distributions. An immediate next step in this research is to examine the performance of linear embedded discrepancy operators after relaxing these restrictions on the random interaction matrices.

## Appendix A. Model Conversion

A system of *S* coupled ordinary differential equations can sometimes be converted (decoupled) to a system of *s* differential equations, where s<S, without loss of information. Possible structures of the resultant set, comprising *s* equations, motivates the functional form of the proposed model discrepancy here. This appendix briefly reviews two methods of model conversion, and what the application of each method yields in the GLV context. For more information about these types of model conversion, or exact model reduction, see [33,42].

### Appendix A.1. Algebraic Method

In [43], Harrington and van Gorder present a method to algebraically convert systems of coupled differential equations from one form to another. As an example from that paper, consider the Lorenz system of three ODEs:

(A1a) $$\begin{array}{cc} \dot{x} & =a(y-x) \end{array}$$

(A1b) $$\begin{array}{cc} \dot{y} & =x(b-z)-y \end{array}$$

(A1c) $$\begin{array}{cc} \dot{z} & =xy-cz. \end{array}$$

Through algebraic substitutions, this can be converted to a single third-order nonlinear differential equation in only the variable *x* and its derivatives. After substituting expressions for *z* and *y* in terms of *x* and its derivatives, we have:

(A2) $$\left(\frac{d}{dt}+c\right)\left(b-\frac{1}{ax}\left(\ddot{x}+\left(1+a\right)\dot{x}+ax\right)\right)-x\left(\frac{\dot{x}}{a}+x\right)=0.$$

In this example, variables *y* and *z* have been exchanged for derivatives of *x*.

In the GLV setting, we can perform a similar exchange. For example, consider the following system for *x* and *y*:

(A3a) $$\begin{array}{cc} \dot{x} & =r_{1}x+\left(a_{11}x+a_{12}y\right)x \end{array}$$

(A3b) $$\begin{array}{cc} \dot{y} & =r_{2}y+\left(a_{21}x+a_{22}y\right)y. \end{array}$$

This is in fact equivalent to the following single differential equation for *x*:

(A4) $$\begin{array}{cc} \left(\frac{d}{dt}\right)\left(\frac{1}{a_{12}}\left(\frac{1}{x}\left(\dot{x}-r_{1}x\right)-a_{11}x\right)\right) & =\\ \frac{r_{2}}{a_{12}}\left(\frac{1}{x}\left(\dot{x}-r_{1}x\right)-a_{11}x\right) & +\left(a_{21}x+\frac{a_{22}}{a_{12}}\left(\frac{1}{x}\left(\dot{x}-r_{1}x\right)-a_{11}x\right)\right). \end{array}$$

Equation (A4) can also be written more compactly as

(A5) $$\dot{z}=r_{2}z+\left(a_{21}x+a_{22}z\right)z,$$

where

(A6) $$z=\frac{1}{a_{12}}\left(\frac{1}{x}\left(\dot{x}-r_{1}x\right)-a_{11}x\right).$$

While this single differential equation is quite messy, it is now written entirely in terms of *x* and its derivatives.

### Appendix A.2. Memory Method

Similarly, the Mori–Zwanzig approach to model reduction makes an exchange, but here, variables may be exchanged for time history, or memory, of the remaining variables. Again, a simple example starts with a two-variable system:

(A7) $$\begin{array}{cc} \frac{dx}{dt} & =f\left(x,y\right)+\alpha \left(x,y\right)\frac{dU}{dt} \end{array}$$

(A8) $$\begin{array}{cc} \frac{dy}{dt} & =g\left(x,y\right)+\beta \left(x,y\right)\frac{dV}{dt}, \end{array}$$

where U,V are noise processes. This system of two equations can be converted to one by introducing the memory kernel *K*:

(A9) $$\frac{dx(t)}{dt}=\bar{f}\left(x\left(t\right)\right)+\int_{0}^{t}K\left(x(t-s),s\right)ds+n\left(x(0),y(0),t\right)$$

where $\bar{f}\left(x\left(t\right)\right)$ represents a Markovian term that depends only on the current state of *x*, the integral $\int_{0}^{t}K\left(x(t-s),s\right)ds$ depends on the entire history of *x* between 0 and *t*, and the final term $n\left(x(0),y(0),t\right)$ satisfies an auxiliary equation. Further details about this process are provided in [44].

The analogue of a Mori–Zwanzig type process in the GLV setting (S=2, s=1) yields:

(A10) $$\dot{x}=r_{1}x+\left(a_{11}x+a_{12}\chi \right)x$$

where

(A11) $$\chi =\int_{0}^{t}r_{2}z+\left(a_{21}x+a_{22}z\right)z$$

and *z* is defined as in the previous subsection. Note that, like the algebraic reduction, we now have a single differential equation in terms of *x*. In this case, the variable *y* is exchanged for the memory of *x*.
